# Triptorelin relieves lower urinary tract symptoms in Chinese advanced prostate cancer patients: a multicenter, non-interventional, prospective study

**DOI:** 10.1186/s12894-018-0337-4

**Published:** 2018-03-27

**Authors:** Le-Ye He, Ming Zhang, Zhi-Wen Chen, Jian-Lin Yuan, Ding-Wei Ye, Lu-Lin Ma, Hui Wei, Jiang-Gen Yang, Shan Chen, Ben Wan, Shu-Jie Xia, Zhi-Liang Weng, Xiang-Bo Kong, Qiang Wei, Feng-Shuo Jin, Xiang-Hua Zhang, Wei-Qing Qian, Shu-Sheng Wang, Ying-He Chen, Hong-Shun Ma, Ying-Hao Sun, Xu Gao

**Affiliations:** 1 Department of Urology, Third Xiangya Hospital, Central South University, Changsha, Hunan China; 2 Department of Urology, the Second Hospital of Jilin University, Changchun, China; 3 Department of Urology, Southwest Hospital, Third Military Medical University, Chongqing, China; 4 Department of Urology, Xijing Hospital, the Fourth Military Medical University, Xi’an, China; 5 Department of Urology, Fudan University Shanghai Cancer Center, Shanghai, China; 6 Department of Urology, Peking University Third Hospital, Beijing, China; 7 Department of Urology, Shenzhen Zhongshan Urological Hospital, Shenzhen, China; 8 Department of Urology, Shenzhen People’s Hospital, The Second Clinical Medical College of Ji’nan University, Shenzhen, China; 9 Department of Urology, Beijing Tongren Hospital Capital Medical University, Beijing, China; 10 Department of Urology, Beijing Hospital of the Ministry of Health, Beijing, China; 11 Department of Urology, Shanghai First People’s Hospital Affiliated to Shanghai Jiaotong University, Shanghai, China; 12 Department of Urinary Surgery, the First Affiliated Hospital of Wenzhou Medical College, Wenzhou, China; 13 Department of Urology, China-Japan Union Hospital, Jilin University, Changchun, China; 14 Departmentof Urology, West China Hospital, Sichuan University, Chengdu, Sichuan China; 15 Department of Urinary Surgery, Institute of Surgery Research, Daping Hospital, Third Military Medical University, Chongqing, China; 16 Department of Urology, Shougang Hospital of Peking University, Beijing, China; 17 Department of Urology, Huadong Hospital, Fudan University, Shanghai, China; 18 Department of Urology, Guangdong Provincial Hospital of Chinese Medicine, Guangzhou University of Chinese Medicine, Guangzhou, China; 19 Department of Urology, Second Affiliated Hospital of Wenzhou Medical College, Wenzhou, China; 20 Department of Urology, Tianjin First Central Hospital, Tianjin, China; 21 Department of Urology, Changhai Hospital, Second Military Medical University, 168 Changhai Road, Shanghai, 200433 China

**Keywords:** Prostate cancer, Lower urinary tract symptoms (LUTS), Prevalence, International prostate symptoms score (IPSS), Triptorelin

## Abstract

**Background:**

Although triptorelin is increasingly used in China for biochemical castration, its effects on primary prostate cancer symptoms remain unclear. This study aimed to assess the prevalence of lower urinary tract symptoms (LUTS) in Chinese prostate cancer patients and the effectiveness of triptorelin on LUTS.

**Methods:**

In this 48-week multicenter, non-interventional, prospective study, we enrolled patients with locally advanced or metastatic prostate cancer. Patients received triptorelin (15 mg) intramuscularly at baseline and at weeks 12, 24, and 36 with symptom assessment using the International Prostate Symptoms Score (IPSS). The primary endpoints were the prevalence of LUTS at baseline per IPSS categories and the percentage of patients with moderate to severe LUTS (IPSS > 7) at baseline, having at least a 3-point reduction of IPSS score at week 48.

**Results:**

A total of 398 patients were included; 211 (53.0%) and 160 (40.2%) among them had severe and moderate LUTS, respectively. Of the patients with IPSS scores available at baseline and at week 48 (*n* = 213), 81.2% achieved a reduction in IPSS of at least 3 points. Of the patients with moderate to severe LUTS at baseline and IPSS scores available at baseline and at week 48 (*n* = 194), 86.6% achieved a total IPSS reduction of at least 3 points.

**Conclusions:**

The vast majority of Chinese patients with locally advanced or metastatic prostate cancer scheduled to receive triptorelin as part of their standard treatment have severe or moderate LUTS. Triptorelin therapy resulted in sustained improvement of LUTS in these patients.

## Background

The incidence of prostate cancer is increasing in China due to an aging population and changes in diet over the previous decades [1, 2]. Despite considerable improvements in the control of localized disease, one third of patients diagnosed with prostate cancer will progress to an advanced or metastatic stage requiring systemic therapy [3]. Androgen suppression by surgical or medical castration is the treatment of choice for these patients [4, 5], leading to a dramatic involution of the primary cancer and metastases in more than 95% of all cases [4, 5]. With the development of injectable depot formulations of gonadotropin-releasing hormone (GnRH) agonists, chemical castration has become a viable alternative to surgical castration [6].

Triptorelin is an agonist of natural GnRH with increased duration of action and higher affinity for the pituitary receptor compared with the parent compound [7]. It downregulates GnRH receptors and causes a post-receptor desensitization of gonadotrophic cells, resulting in reversible biochemical castration [8]. After initial stimulation, gonadotropin secretion is inhibited by prolonged administration of triptorelin, thereby suppressing testicular function [9].

Triptorelin pamoate (Diphereline®) 3-month depot formulation has been marketed in China since 2010. However, the effect of biochemical castration by triptorelin on the primary symptoms of prostate cancer has not yet been studied in this specific population. Early prostate cancer often does not cause symptoms; although some patients do present with symptoms, the actual incidence of this malignancy is unknown. We carried out this multicenter, non-interventional, prospective study to evaluate the prevalence of lower urinary tract symptoms (LUTS) in Chinese prostate cancer patients scheduled to receive triptorelin and to examine the effectiveness of triptorelin on LUTS.

## Methods

### Patients

This study enrolled patients at 21 centers across China (Appendix 1) between June 2010 and December 2012. Men with locally advanced or metastatic prostate cancer (at least T3 stage), scheduled to receive triptorelin pamoate and mentally and physically fit to answer the questionnaire, were included in this study. The included subjects could have had a history of surgery. Patients were excluded if they had hypersensitivity to triptorelin or one of its excipients, if they were at risk of a serious complication in case of a tumour flare, had received another experimental drug over the last 3 months before the study, had received a luteinizing hormone-releasing hormone (LHRH) analogue in the preceding 6 months, or had a life expectancy < 12 months.

The study protocol was approved by the institutional review boards of each participating center, and the study was performed in compliance with Good Pharmacoepidemiology Practice. All participating centers followed Good Clinical Practice. Written informed consent was obtained from all participants.

### Therapeutic regimen

The decision to prescribe triptorelin was taken by attending physicians before enrolment, and not influenced by participation in the study. Each eligible patient received an intramuscular injection of triptorelin (15 mg) at baseline and at weeks 12, 24, and 36. Patients received concomitant anti-androgen treatment to prevent flares at treatment initiation according to locally accepted guidelines and standard practice.

### Patient evaluation

Urinary symptoms were assessed at baseline, and at 24 and 48 weeks after the start of triptorelin treatment using the International Prostate Symptoms Score (IPSS). The seven symptom questions have a severity scale of 0 to 5, and the total IPSS ranges between 0 and 35. Higher scores reflect greater severity. Total IPSS values of 0, 1-7, 8-19, and 20-35 indicate none, mild, moderate and severe urinary symptoms, respectively. The obstructive (voiding) subscore ranges between 0 and 20, and the irritative (storage) subscore between 0 and 15. PSA (ng/mL) was recorded at baseline and at weeks 24 and 48, only as part of standard care. The subjects’ quality of life (QoL) due to urinary symptoms was assessed by the QoL question of the IPSS.

### Statistical analysis

A sample size of 500 patients was chosen based on feasibility, which would allow estimating the prevalence of LUTS in locally advanced or metastatic prostate cancer patients [(based on a two-sided 95% confidence interval (CI)], with a maximum precision of 0.044 for an estimated prevalence of 0.50. Summary statistics [n, mean, standard deviation (SD)], range, and frequency counts) were provided for demographic and baseline characteristics, including age, height, weight, time since first prostate cancer diagnosis, Gleason score, and indication to start triptorelin treatment. Statistical analyses were pre-specified with the inclusion of all patients with total IPSS baseline data. The full analysis set, i.e. effectiveness population, included all patients who received at least one triptorelin injection with at least one post baseline IPSS assessment. The per-protocol set included all patients from the full set who were not excluded for protocol violation. Unless otherwise specified, all effectiveness results reported herein were based on the full analysis set; for patients who withdrew or were lost to follow-up, the last observation performed was used.

The primary endpoints were the prevalence of LUTS at baseline per IPSS categories and the percentage of patients with moderate to severe LUTS (IPSS > 7) at baseline and having at least 3-point reduction of IPSS score at week 48. Major secondary outcomes were changes from baseline of IPSS total score and obstructive and irritative subscores, changes from baseline of total IPSS categories, changes of PSA and PSA categories from baseline and QoL.

All statistical tests were exploratory and two-sided, at the 5% significance level. Approximate binomial CIs were produced using the Agresti-Coull method. All statistical analyses were performed with the Statistical Analysis System® (SAS®) software version 9.1.3 and 9.2 (SAS Institute, Cary, NC, USA). For the overall analysis based on IPSS categories, the Bhapkars test was used to assess differences between baseline and post-baseline visit distributions. Paired t-test was used to assess if changes from baseline at week 24 and 48 differed from 0 for PSA levels as well as total and each of the IPSS subscores. Pearson’s correlation analysis was performed to assess the association between total IPSS and PSA. Shift tables were also used to describe distribution changes in IPSS categories at week 24 and 48 versus baseline.

## Results

### Patient demographic and baseline characteristics

**Fig. 1:**
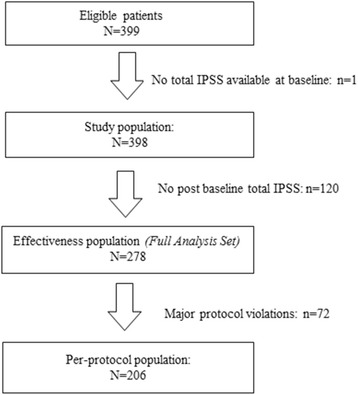
Study flowchart

The study flowchart is shown in Fig. 1. The study intended to enroll 500 locally advanced or metastatic prostate cancer patients scheduled to receive triptorelin, but enrollment was terminated prematurely because of poor recruitment. In total, 399 patients were finally enrolled. One participant was excluded because baseline International Prostate Symptoms Score (IPSS) was not available, and 398 patients were included in the study population. The demographic and baseline characteristics of the study population are shown in Table 1. They were 72.2 ± 8.5 years old, and weighted 65.9 ± 8.9 kg. Slightly more than half (53.1%) of the patients had Gleason scores ≥8; 34.0% and 12.9% had Gleason scores of 7 and ≤ 6, respectively.

**Table 1:** Demographic and baseline characteristics of the study population

Variables	Study Population (*N* = 398)
Age (years)	
Mean ± SD (Range)	72.2 ± 8.5(47, 93)
Height (cm)	
Mean ± SD (Range)	169.2 ± 5.5(145, 186)
Weight (kg)	
Mean ± SD (Range)	65.9 ± 8.9(44, 102)
Gleason score	
N (%)	341 (85.7)
≤ 6	44 (12.9)
7	116 (34.0)
≥ 8	181 (53.1)
TNM stage, n (%)	
T3N0M0	97 (24.4)
T4N0M0	10 (2.5)
T(any)N(any)M+	168 (42.2)
Regional lymph nodes status (N+)	22 (5.5)
Other^a^	101 (25.4)
Time since first prostate cancer diagnosis (years)^b^	
Mean ± SD (Range)	0.1 ± 0.7(0, 8)
Indications to Start Triptorelin Treatment, n (%)	
First line therapy	
Locally advanced prostate cancer	228 (57.3)
Metastatic prostate cancer	134 (33.7)
Others^a^	36(9.1)
Any anti-androgen therapy, n (%)	
Yes	389 (97.7)
Any surgical history, n (%)	
Yes	66 (16.6)
Prior radiotherapy, n (%)	
Yes	16 (4.0)
Prior endocrine therapy for prostate cancer, n (%)	
Yes	14 (3.5)
Any prior medication, n (%)	
Yes	301 (75.6)
Prior endocrine therapy, n (%)	
Yes	299 (75.1)
Bicalutamide	259 (65.1)
Flutamide	40 (10.1)
Goserelin	1 (0.3)
Any concomitant medication, n (%)	
Yes	101 (25.4)
Concomitant endocrine therapy, n (%)	
Yes	95 (23.9)
Bicalutamide	79 (19.8)
Flutamide	17 (4.3)

^a^TNM stage not re-evaluated for disease recurrence after radical treatment

^b^Time since first prostate cancer diagnosis (years) defined as (baseline visit date – date of first prostate cancer diagnosis)/365.25 and rounded to the largest number that was less than or equal to the calculated value

The majority of the patients were diagnosed with T3 (259 patients) or T4 (77 patients) advanced and/or metastatic prostate cancer. The mean time from first prostate cancer diagnosis to baseline was 0.1 ± 0.7 years. Triptorelin was first-line therapy for most patients (90.9%). Two hundred and thirty-nine patients (60.1%) took all four injections of triptorelin and 75 (18.8%) patients took only one injection.

The majority of patients (75.6%) took medications before they entered the study. Bicalutamide was the most commonly used drug. During the study, 101 (25.4%) patients took concomitant medications. Endocrine therapy (*n* = 95; 23.9%), bicalutamide (*n* = 79; 19.8%), Flutamide (*n* = 17; 4.3%), urologicals (*n* = 7; 1.8%), Alfuzosin (*n* = 3; 0.8%), flavoxate hydrochloride (n = 1; 0.3%), Tamsulosin (n = 1; 0.3%), terazosin (*n* = 1; 0.3%), and tolterodine L-tartrate (*n* = 1; 0.3%) were also administered. Among the 398 patients assessed, 66 (16.6%) had a history of surgery, mostly radical or transurethral prostatectomies.

IPSSs during the treatment were missing for 120 participants (30.1%), and 278 patients were included in the full analysis set. There were 72 cases of major protocol violations, and 206 patients were included in the per-protocol set.

### Primary outcome measures

#### Prevalence of LUTS

In the study population, 211 (53.0%), 160 (40.2%), and only 26 (6.5%) patients had severe, moderate and mild LUTS at baseline, respectively.

Effectiveness of triptorelin therapy in reducing total IPSS.

Effectiveness of triptorelin therapy in reducing total IPSS is shown in Table 2. In the full analysis population, 277 patients had LUTS at baseline, including 213 with total IPSS available at week 48. The vast majority (81.2%; 95%CI 75.4, 85.9) achieved an IPSS reduction of at least 3 points with triptorelin therapy at week 48. Moreover, 255 (91.7%) patients had moderate to severe LUTS at baseline, including 194 with total IPSS available at week 48, of which 168 (86.6%; 95%CI 81.0, 90.7) patients had a total IPSS reduction of at least 3 points after 48 weeks of triptorelin therapy. Furthermore, 212 (83.1%) patients with moderate to severe LUTS at baseline had non-operated prostate cancer. At week 24, 57.1% (145/254) of the non-operated prostate cancer patients achieved a total IPSS reduction of at least 3 points, which further increased to 70.1% (136/194; 95%CI 63.3, 76.1) at week 48.

**Table 2:** Effectiveness of triptorelin therapy in reducing total IPSS (full analysis population)

		LUTS at baseline (*N* = 277) and IPSS data at week 48 (*N* = 213)	Moderate to severe LUTS at baseline (*N* = 255) and IPSS data at week 48 (*N* = 194)	Moderate to severe LUTS at baseline with non-operated prostate cancer (*N* = 212) and IPSS data at week 48 (*N* = 194)
LUTS at baseline and ≥ 3 point reduction in IPSS	N (%)	173 (81.2)	168(86.6)	136 (70.1)
	95% CI	(75.4, 85.9)	(81.0, 90.7)	(63.3, 76.1)

### Secondary outcome measures

#### IPSS total score, obstructive and irritative subscores

**Fig. 2:**
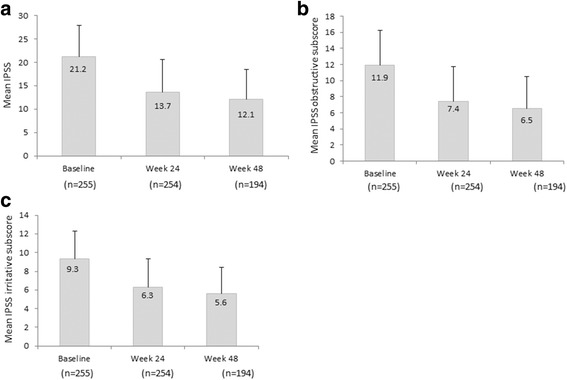
Mean change from baseline at weeks 24 and 48 in patients with moderate to severe LUTS and prostate cancer receiving triptorelin therapy. **a** Total IPSS, (**b**) IPSS obstructive (voiding) subscore, (**c**) IPSS irritative (storage) subscore. Error bars represent standard deviations. IPSS, International Prostate Symptoms Score; LUTS, lower urinary tract symptoms

The mean total IPSS was 21.2 ± 6.7 at baseline for 255 patients who had moderate to severe LUTS at baseline, which decreased to 13.7 ± 6.9 at week 24, with a mean change of − 7.5 ± 7.2 from baseline (95%CI, − 8.4 to − 6.6) (Fig. 2a). The mean total IPSS further decreased to 12.1 ± 6.4 at week 48, with a mean change of − 9.0 ± 7.3 from baseline (95%CI, − 10 to − 8.0). The mean baseline IPSS obstructive subscore for patients with moderate to severe LUTS at baseline was 11.9 ± 4.3, which was reduced to 7.4 ± 4.3, with a mean change of − 4.5 ± 4.7 from baseline at week 24 (95%CI, − 5.0 to − 3.9) (Fig. 2b). The mean IPSS obstructive subscore was further reduced to 6.5 ± 4.0, with a mean change of − 5.3 ± 4.7 from baseline at week 48 (95%CI, − 6.0 to − 4.6). The mean baseline IPSS irritative subscore for patients with moderate to severe LUTS at baseline was 9.3 ± 3.0, which declined to 6.3 ± 3.0, with a mean change of − 3.0 ± 3.2 from baseline at week 24 (95%CI, − 3.4 to − 2.6) (Fig. 2c). The mean IPSS irritative subscore was further reduced to 5.6 ± 2.8, with a mean change of − 3.7 ± 3.3 from baseline at week 48 (95%CI, − 4.2 to − 3.2).

#### Changes in total IPSS categories

**Fig. 3:**
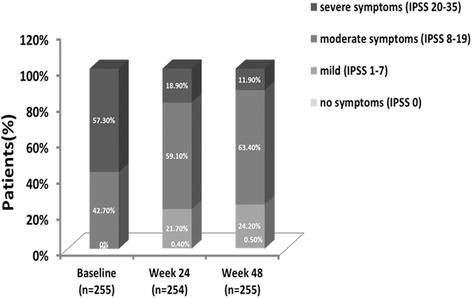
Proportions of triptorelin-treated patients with no, mild, moderate or severe LUTS at baseline, and at weeks 24 and 48. LUTS, lower urinary tract symptoms

In the full analysis population, 146 (57.3%) patients had severe symptoms at baseline, which decreased to 18.9% at week 24, and 11.9% at week 48 (Fig. 3). More than 20% of patients with moderate to severe LUTS at baseline had improvements to mild LUTS after triptorelin therapy (21.7% and 24.2% at weeks 24 and 48, respectively). At week 48, 12/65 (18.5%) patients with moderate symptoms at baseline improved to mild symptoms, 68/97 (70.1%) patients with severe symptoms at baseline improved to moderate symptoms, and 8/97 (8.3%) patients improved to mild symptoms. A similar trend was observed at week 24.

#### Changes in PSA levels

**Fig. 4:**
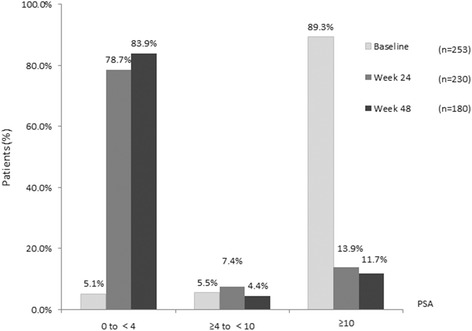
PSA levels (ng/mL) at baseline and study visits by PSA category

At baseline, 89.3% of patients had PSA levels ≥10 ng/mL, 5.1% with PSA 0 to < 4 ng/mL, and 5.5% with PSA ≥4 to 10 ng/mL. At week 48, most (83.9%) patients had PSA levels from 0 to < 4 ng/mL while only 11.7% of patients had PSA levels ≥10 ng/mL (Fig. 4). Mean PSA change from baseline to week 24 and 48 was − 286.6 ± 1095.7 ng/mL (95%CI, − 429.2 to − 143.9) and − 259.9 ± 986.2 ng/mL (95%CI, − 405.3 to − 114.4), respectively. All patients who had a PSA level of ≥4 to < 10 ng/mL at baseline had their PSA revert back to < 4 ng/mL from week 24 except one patient who had an increased PSA level at week 24. Pearson’s correlation analysis revealed no correlation between PSA changes and total IPSS changes from baseline at weeks 24 (rho = − 0.046; *P* = 0.532) and 48 (rho = 0.087; *P* = 0.289).

#### QoL

At baseline, the majority of prostate cancer patients with urinary symptoms were unhappy (30.6%), mostly dissatisfied (31.8%), or terribly dissatisfied (14.9%) with their QoL; only 1.2% of the assessed patients were pleased and 5.1% mostly satisfied with their QoL (Table 3). After 48 weeks of treatment with triptorelin, 10.8% of patients were delighted, with 12.9% pleased, 30.4% mostly satisfied, and 26.8% equally satisfied and dissatisfied with their QoL. Only 5.7% of the tested patients were unhappy, with 12.9% mostly dissatisfied; only 1 patient (0.5%) was terribly dissatisfied with their QoL.

**Table 3:** Quality of life of patients with prostate cancer and moderate to severe LUTS by visit

	Baseline	Week 24	Week 48	Last Available Visit
No.	255	254	194	255
Quality of life due to urinary symptom, n (%)				
0 – delighted	0	13 (5.1)	21 (10.8)	21 (8.2)
1 – pleased	3 (1.2)	35 (13.8)	25 (12.9)	32 (12.5)
2 – mostly satisfied	13 (5.1)	54 (21.3)	59 (30.4)	72 (28.2)
3 – mixed – about equally satisfied and dissatisfied	42 (16.5)	73 (28.7)	52 (26.8)	72 (28.2)
4 – mostly dissatisfied	81 (31.8)	61 (24.0)	25 (12.9)	43 (16.9)
5 – unhappy	78 (30.6)	12 (4.7)	11 (5.7)	12 (4.7)
6 – terrible	38 (14.9)	6 (2.4)	1 (0.5)	3 (1.2)

*IPSS* international prostate symptom score, *LUTS* lower urinary tract symptoms

## Discussion

This 48-week multicenter, non-interventional, prospective study assessed the baseline LUTS rates of patients with locally advanced or metastatic prostate cancer. Of these patients, 93.2% had severe to moderate LUTS, a noticeably higher proportion than in a Belgian population study (61.5%) [10] and a recent observational grouped analysis (52.1%) [11]. Total mean IPSS and mean irritative /obstructive scores were also higher in our study than those reported in the Belgian study (total mean IPSS: 21.1 vs. 14.0; mean irritative score: 9.3 vs. 6.5; mean obstructive score: 11.9 vs. 7.5) [10]. These findings suggest that more attention should be focused on the high prevalence of LUTS in Chinese patients with prostate cancer, and highlight the differences in severity of LUTS between Chinese and European populations.

Androgens act via the androgen receptor to regulate the proliferation of cells in the prostate as well as prostate cancer cells, and the effectiveness of androgen deprivation in treating prostate cancer is clear evidence for their importance in driving disease progression [12]. Triptorelin is a GnRH agonist that results in reversible biochemical castration, and its role in treating patients with prostate cancer is well established [13, 14]; however, its efficacy on the primary symptoms of prostate cancer, such as LUTS, has not yet been extensively studied [9, 11, 15, 16]. Our study revealed that Chinese patients with locally advanced or metastatic prostate cancer scheduled to receive triptorelin as part of standard treatment achieved clinically meaningful improvements in LUTS (IPSS reduction > 3) from baseline, maintained throughout the study. Most patients with moderate to severe LUTS at baseline had a total IPSS reduction of at least 3 points after 48 weeks of triptorelin therapy (86.6%). Triptorelin was also effective in patients with non-operated prostate cancer; most of them achieved a total IPSS reduction of at least 3 points at week 48 (70.1%), which was seen as early as week 24 in more than half (57.1%) of the patients.

At weeks 24 and 48, improvements from baseline in mean total IPSS were achieved for patients with moderate to severe LUTS at baseline (21.2, 13.7 and 12.1, respectively). Although there is no direct comparison with other GnRH agonists for efficacy on LUTS for prostate cancer patients, the reductions in total IPSS appear to be similar to those reported among patients receiving goserelin in previous studies [17]. Additionally, there were improvements in mean IPSS irritative and obstructive subscores at week 48 in these patients. Improvements in LUTS were associated with QoL benefits for patients with locally advanced or metastatic prostate cancer. At baseline, the majority of patients with moderate to severe LUTS were unhappy or mostly dissatisfied with their QoL due to urinary symptoms. After 48 weeks of treatment with triptorelin, more than half of patients were delighted, pleased, or satisfied with their QoL.

Consistent with previous studies [13, 14], decreases in PSA levels from baseline to weeks 24 and 48 were observed with triptorelin therapy in this study. In patients with moderate to severe LUTS at baseline who had PSA levels ≥10 ng/mL at baseline (89.3%), PSA decreased to < 4 ng/mL by the end of the study (83.9%). However, we found no correlation between PSA change from baseline and total IPSS change from baseline.

The present analysis reported a high prevalence of LUTS for prostate cancer patients in China and confirmed the efficacy of triptorelin on LUTS for Chinese patients. However, the present study had limitations. First, it failed to recruit the intended number of participants, and enrolment was terminated prematurely. In addition, nearly one third of patients (30.1%, *n* = 120) had no post baseline total IPSS and thus were excluded from the full analysis. Meanwhile, some medications administered concomitantly with triptorelin might affect LUTS, biasing our analysis. The prevalence of LUTS reported in this study may be higher than in routine clinical practice. Nevertheless, the severity of LUTS and the high rate of advanced prostate cancer reported in this study should serve to increase our awareness of this disease, and highlight the importance of its timely diagnosis and management.

## Conclusions

In conclusion, nine out of ten Chinese patients with locally advanced or metastatic cancer had severe or moderate LUTS at baseline, which negatively impacts their QoL. Triptorelin therapy improved LUTS in these prostate cancer patients; these effects were maintained during the study, leading to clinically meaningful improvements in QoL.

## Appendix

**Table 4:** List of participating institutions

NO.	Hospital	Investigator	Location
1	Changhai Hospital, Second Military Medical University	Ying-Hao Sun	Shanghai
2	Third Xiangya Hospital, Central South University	Le-Ye He	Changsha
3	The Second Hospital of Jilin University	Ming Zhang	Changchun
4	Southwest Hospital, Third Military Medical University	Zhi-Wen Chen	Chongqing
5	Xjiing Hospital, The Fourth Military Medical University	Jian-Lin Yuan	Xi’an
6	Fudan University Shanghai Cancer Center	Ding-Wei Ye	Shanghai
7	Peking University Third Hospital	Lu-Lin Ma	Beijing
8	Shenzhen Zhongshan Urological Hospital	Hui Wei	Shenzhen
9	Shenzhen People’s Hospital, The Second Clinical Medical College of Ji’nan University	Jiang-GenYang	Shenzhen
10	Beijing Tongren Hospital Capital Medical University	Shan Chen	Beijing
11	Beijing Hospital of the Ministry of Health	Ben Wan	Beijing
12	Shanghai First People’s Hospital Affiliated to Shanghai Jiaotong University	Shu-Jie Xia	Shanghai
13	The First Affiliated Hospital of Wenzhou Medical College	Zhi-Liang Weng	Wenzhou
14	China-Japan Union Hospital, Jilin University	Xiang-Bo Kong	Changchun
15	West China Hospital, Sichuan University	Qiang Wei	Chengdu
16	Daping Hospital, Third Military Medical University	Feng-Shuo Jin	Chongqing
17	Shougang Hospital of Peking University	Xiang-Hua Zhang	Beijing
18	Huadong Hospital, Fudan University	Wei-Qing Qian	Shanghai
19	Guangdong Provincial Hospital of Chinese Medicine, Guangzhou University of Chinese Medicine	Shu-Sheng Wang	Guangzhou
20	Second Affiliated Hospital of Wenzhou Medical College	Ying-He Chen	Wenzhou
21	Tianjin First Central Hospital	Hong-Shun Ma	Tianjin

## Abbreviations

CI: Confidence interval
GnRH: Gonadotropin-releasing hormone
IPSS: International Prostate Symptoms Score
LHRH: Luteinizing hormone-releasing hormone
LUTS: Lower urinary tract symptoms
QoL: Quality of life
SAS: Statistical Analysis System
SD: Standard deviation
